# Clinical Evaluation of Placement of Implant by Flapless Technique Over Conventional Flap Technique

**DOI:** 10.1007/s12663-019-01218-9

**Published:** 2019-03-30

**Authors:** T. K. Divakar, Sundaram Gidean Arularasan, M. Baskaran, I. Packiaraj, N. Dhineksh Kumar

**Affiliations:** 1 Department of Oral and Maxillofacial Surgery, Rajas Dental College and Hospital, Tirunelveli, India; 2 Department of Dental and Oral surgery, Unit -II, Christian Medical College and Hospital, Vellore, India; 3 Gem Head and Neck Hospital, Palayamkottai, Tirunelveli, India

**Keywords:** Conventional flap, Flapless, Implant, Marginal bone loss

## Abstract

**Aim:**

The aim of this study was to compare the clinical advantages of flapless implant surgery over conventional flap technique of implant placement by assessing the marginal bone loss in 1 month, 2 months and 3 months postoperatively, pain assessment, number of analgesics taken by the patients postoperatively and the postoperative swelling between two groups.

**Materials and Methods:**

This study was conducted at Department of Oral and Maxillofacial Surgery, Rajas Dental College and Hospital, Tirunelveli. The patients were assigned randomly to one of the two groups—flap (ten patients) or flapless (ten patients). Digital IOPARs were taken postoperatively. The parameters assessed were marginal bone loss (interproximal bone height), pain assessment by a 10-cm visual analog scale, swelling assessment by modification of tape measuring method by Gabka and Matsumara and the number of analgesics tablets taken every postoperative day from the day of surgery to 6 days after surgery.

**Results and Statistics:**

Descriptive statistics was done by calculating measures of central tendency (mean) and measures of dispersion (standard deviation) for all the parameters. Inferential statistics were done by unpaired Student’s *t* test to compare the mean difference between the two groups. The results of this study showed that the mean difference in the bone loss for baseline to the third month for the flap group was 0.34 ± 0.05 and for the flapless group was 0.03 ± 0.004 (*p* = 0.000***). Pain assessment by visual analog scale was statistically significant in all the 5 postoperative days indicating a better patient compliance in the flapless group and there was no statistical difference in the level of swelling between these two groups.

**Conclusion:**

Within the limitations of this study, it can be concluded that flapless implant surgery results in lesser loss of marginal bone and also results in better patient comfort; however, proper patient selection and technique is essential for a successful flapless implant surgery.

## Introduction

The problem of missing teeth has troubled mankind ever since times immemorial. With advancements in material sciences and improvement in understanding of occlusion and the gnathostomatic system, better modalities of tooth replacement came into existence. The developments were all concerned with the three primary goals of comfort, function and esthetics, and any development which benefited in these goals was popularized.

Implant dentistry has evolved from a traditional conventional flap therapy to a highly esthetic-driven discipline. Consequently, clinicians have sought to implement techniques to shorten the treatment with methods such as immediate placement of implants at the time of extraction, immediate loading and flapless surgical procedures.

The flapless surgical approach was introduced in the late 1970s by Ledermann [1] to overcome the bone resorption process. Studies comparing the crestal bone height using the flapless and the flap surgical techniques are minimal. The purpose of this study is to compare the clinical advantages of flapless implant surgery over conventional flap technique of implant placement.

## Materials and Methods

This study was conducted at Department of Oral and Maxillofacial Surgery, Rajas Dental College and Hospital, Tirunelveli. The institutional scientific review board and ethical committee approved the protocol of this nonrandomized clinical prospective study. The study population comprised of flapless implant surgery group and conventional open flap implant surgery group. Both the groups had ten patients each. Patients who were above 18 years of age with partially edentulous jaw requiring single or multiple tooth replacement with a minimum of 5 mm of bone width and 8 mm height at the implant site, who were willing to comply with the treatment regimen and had not undergone extraction of not less than 6 months at the extraction site were included in the study. Patients with systemic diseases contraindicating any type of surgery, on bisphosphonates, any evidence of pathology or active diseases of the implant bed (e.g., residual cysts) and atrophy requiring bone regeneration in both width and height were excluded from the study.

## Surgical Procedure

**Fig. 1:**
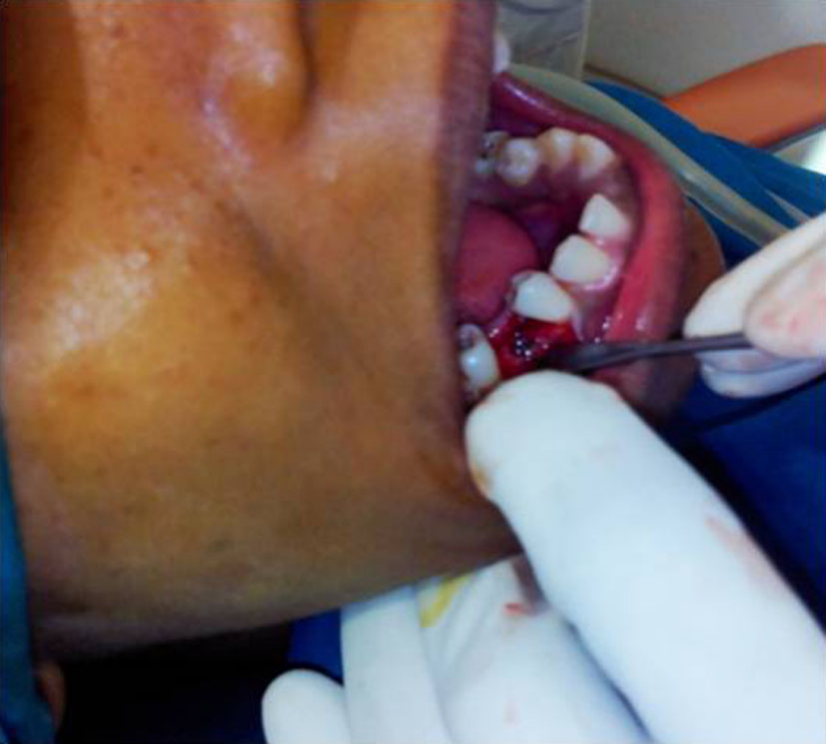
Flap elevation and implant placement—flap group

**Fig. 2:**
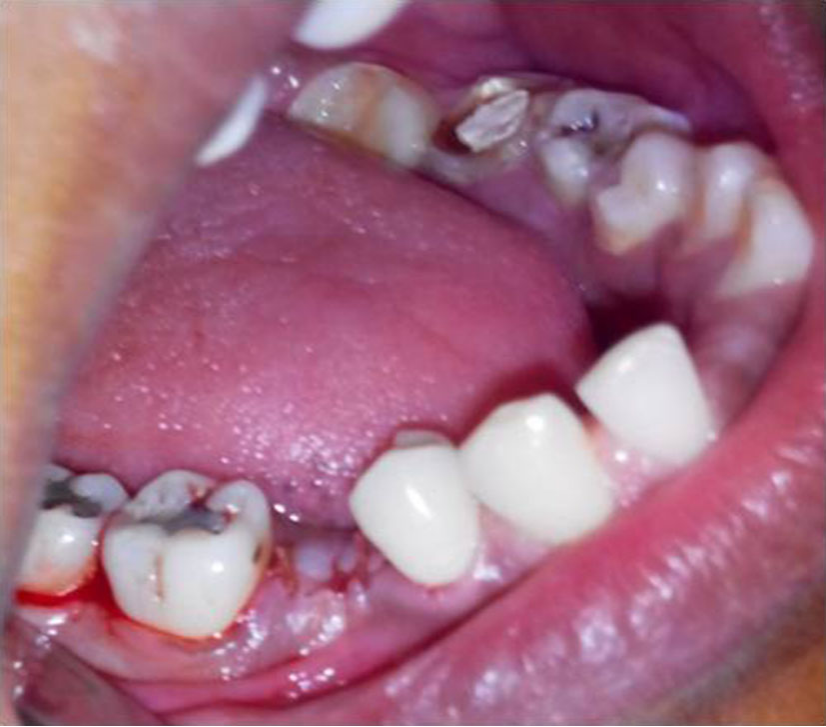
Sutured with 3–0 Vicryl—flap group

The surgical field was prepared and the implant site was anesthetized with 2% lidocaine with 1:80,000 epinephrine. In conventional flap group, midcrestal incision was placed with sulcular extensions to adjacent teeth on either side with a Bard-Parker blade no. 15 (Fig. 1) and then a full-thickness mucoperiosteal flap was raised. Initial entry was made with a no. 5 round bur followed by pilot drill to the required depth. Then, successive drills were made till the required diameter is achieved. The implant was then placed into the prepared site and the flaps were closed with interrupted sutures (3–0 Vicryl, Ethicon) (Fig. 2).

**Fig. 3:**
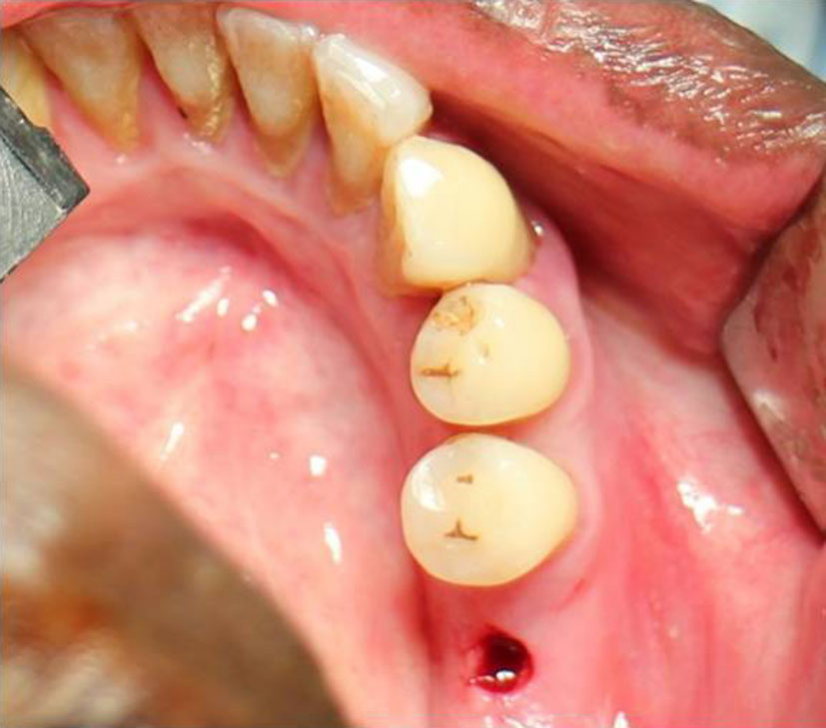
Soft tissue punch—flapless group

**Fig. 4:**
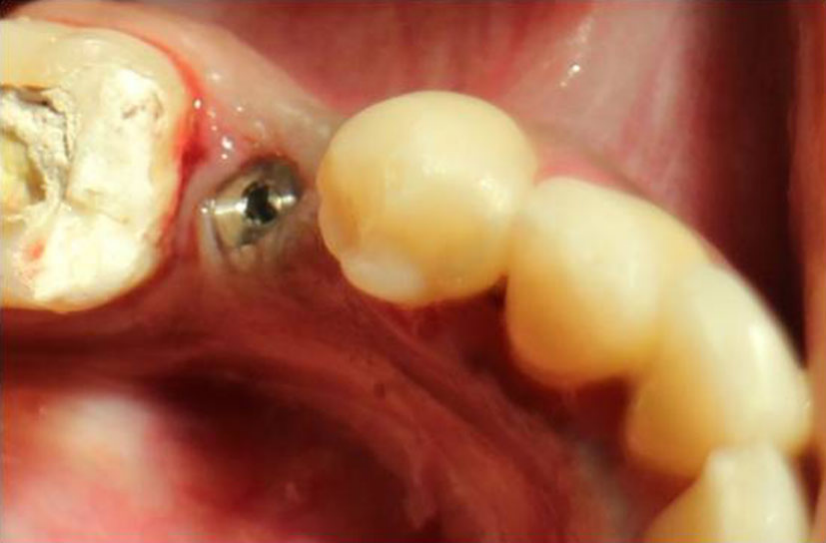
Flapless implant placement with cover screw

In the flapless group, a surgical guide stent was fabricated from the diagnostic models and a hole was drilled in the stent opposite to the missing tooth indicating the position of the implant. Soft tissue punch was used to make initial entry into the soft tissue (Fig. 3). The diameter of the soft tissue punch was decided according to the implant to be placed finally after osteotomy. Soft tissue punch available in three different sizes: 3 mm, 4 mm and 5 mm, was used in this study. After the soft tissue punch was made, the pilot drill was used to reach the required depth. Then, it was followed by incremental drills till the desired diameter is achieved. Once the osteotomy was finished, implant was placed inside the bone (Fig. 4).

Patients in both the groups underwent a two-stage implant placement procedure. In stage I surgery, implants with cover screw were placed and then left for 3 months for bone healing. In stage II surgery, for flap group the crestal incision was placed and the cover screw was exposed. After the removal of cover screw, healing cap was placed and then patient was referred to Department of Prosthodontics for further prosthetic rehabilitation. In the flapless group, tissue punch was used to expose the cover screw and the healing cap was placed for 1 week. The patient was then referred to Department of Prosthodontics for prosthetic rehabilitation.

## Postsurgical Assessment

Digital IOPARs were taken postoperatively. All the patients were given amoxicillin 500 mg three times daily for 5 days and ibuprofen 400 mg which was informed to patient to take only if needed. Patients were recalled on the postoperative second day and seventh day for swelling assessment. Recall appointments were made at 1 month, 2 months and 3 months postoperatively. The parameters assessed were marginal bone loss (interproximal bone height), pain assessment by a 10-cm visual analog scale, swelling assessment by modification of tape measuring method by Gabka and Matsumara and the number of analgesics tablets taken every postoperative day from the day of surgery to 6 days after surgery which were recorded.

## Marginal Bone Loss

**Fig. 5:**
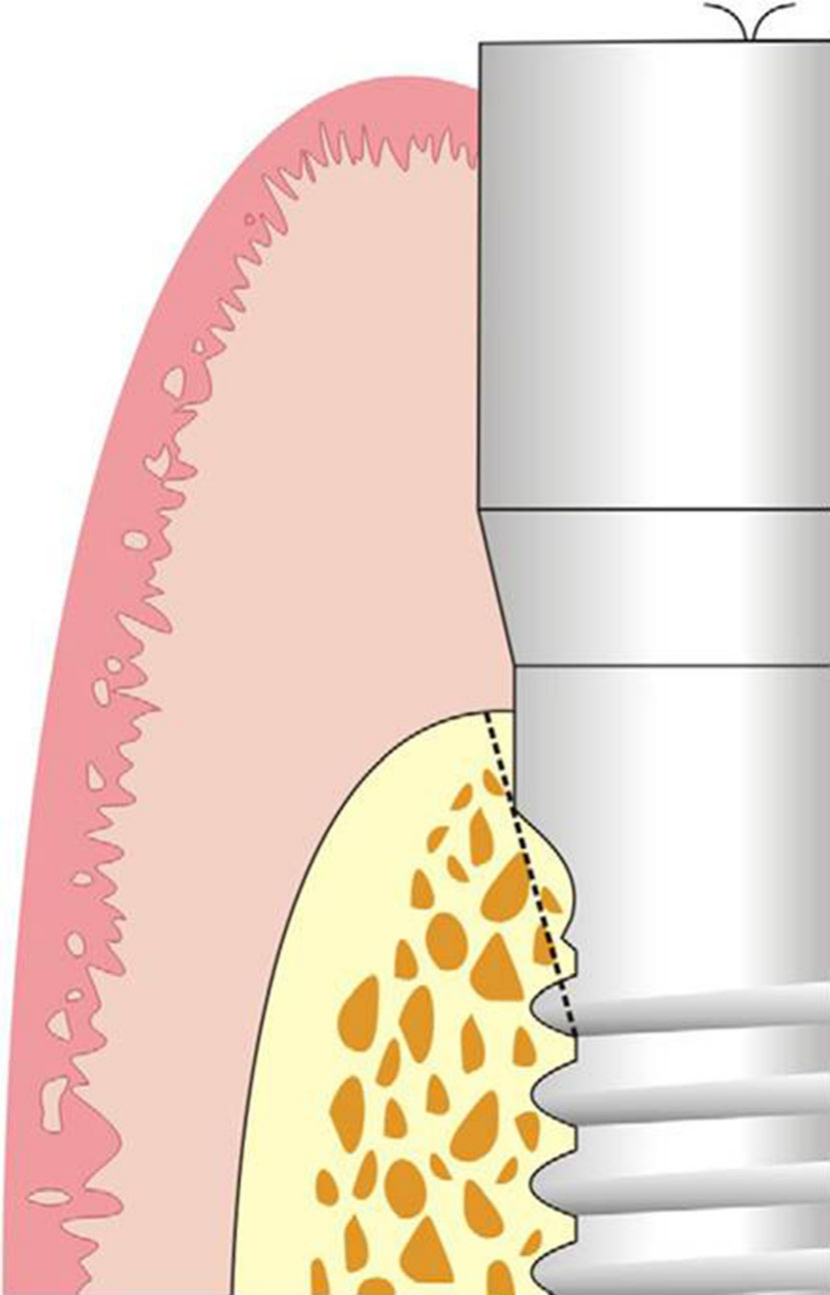
Marginal bone loss measurement

**Fig. 6:**
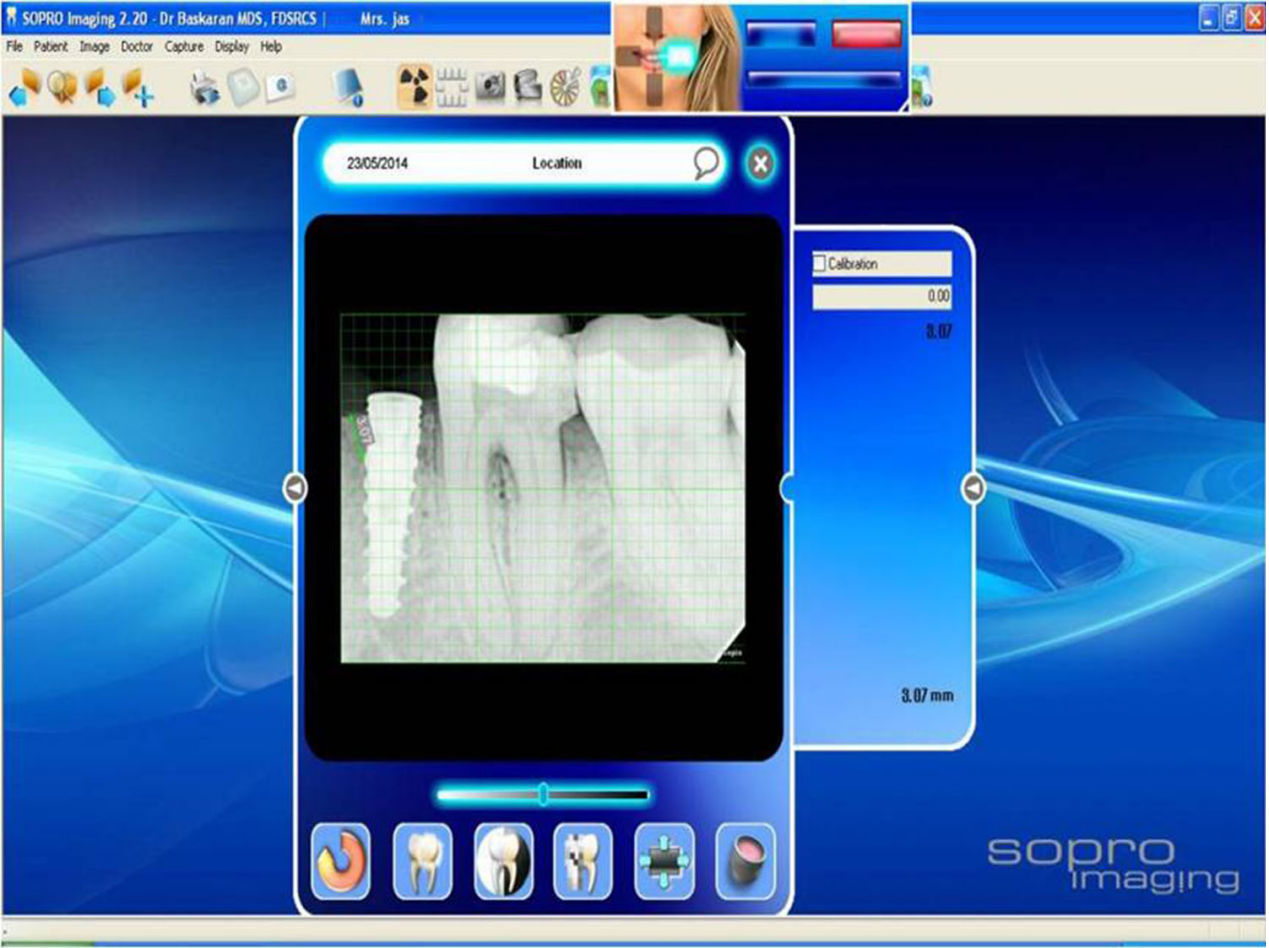
SORPO imaging software

**Fig. 7:**
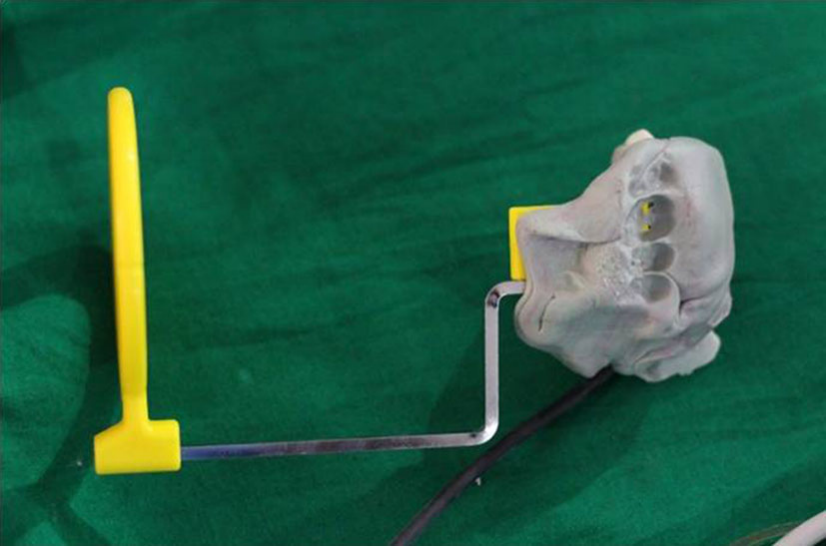
Radiographic standardization with putty index

It was measured by measuring the interproximal height of bone which was defined as the distance measured between the apical ends of the first thread of the implant to the most coronal point of the interproximal crestal bone [2] (Fig. 5). This value was recorded using the digital IOPARs taken. This parameter was recorded at baseline (immediately after implant placement), 1 month, 2 months and 3 months postoperatively. The paralleling cone technique was used to standardize the radiographs. All radiographs taken were digital radiographs. The SORPO imaging software 2.2 (Acteon Group, France) was used to make all the measurements on the radiographs (Fig. 6). Measurements were taken using a line tool. The marginal bone loss was measured for each implant placed at baseline (immediately after implant placement), 1 month, 2 months and 3 months postoperatively. The difference in the bone height was measured for each time period for the flapless and the conventional flap groups. The radiographs were standardized to access the position of the implant and marginal bone loss measurements. Crestal bone height measurements were taken using paralleling cone technique. The sensor was placed in a holder and positioned in the mouth parallel to the long axis of the implant. The parallelism between the X-ray sensor and the tube was achieved by using XCP-DS digital sensor positioning system for SORPO digital X-ray systems by Dentsply Rinn (Illinois, USA). This system consisted of a plastic ring on which the X-ray tube was supported when X-ray was being taken. This entire setup results in the X-ray being projected being right angles to the X-ray sensor and the implant under investigation. The ring was joined by a stem to the X-ray sensor support and was fitted with a clip on which the patient bites to keep the system stable. In order to reproduce the position of the X-ray taken at baseline, the patient was asked to bite on putty index placed in the clip while taking the baseline X-ray (Fig. 7). This putty index was used for every other three recall visits so that the position of the X-ray tube, sensor and the implant angulation become reproducible making the system standardized.

## Pain and Analgesic Assessment

**Fig. 8:**
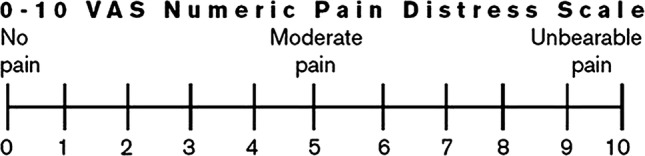
VAS

The patients were requested to complete two sheets of table every evening for 1 week from day of surgery (D0) to 6 days after surgery to report the level of pain and the number of analgesics taken. The patient had to evaluate the pain on a 10-cm visual analog scale (VAS) [21] ranging from 0 (no pain) to 10(unbearable pain) (Fig. 8).

## Swelling Assessment

**Fig. 9:**
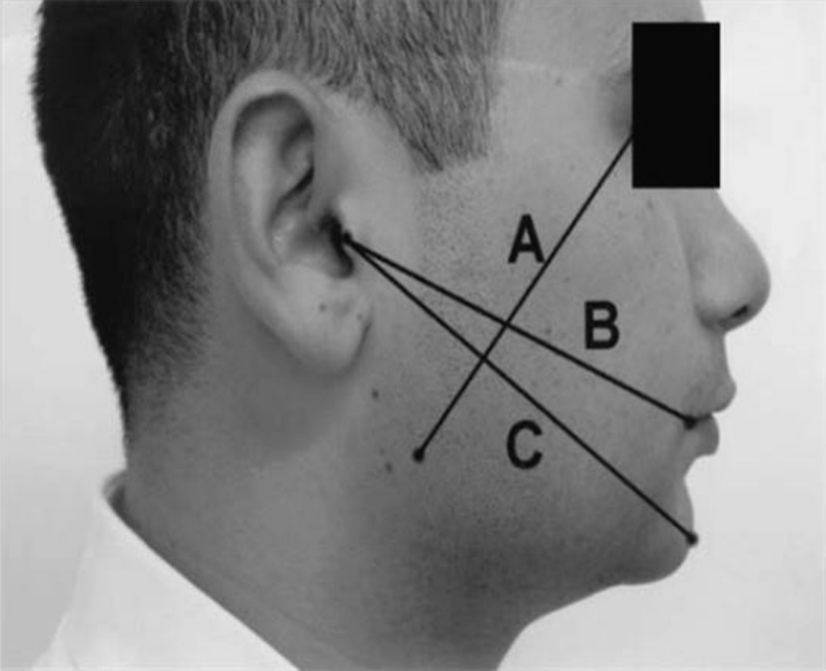
Swelling assessment

The level of facial swelling was determined by a modification of tape measuring method described by Gabaka and Matsumara [3]. Three measurements were taken between five reference points: tragus, soft tissue pogonion, lateral corner of the eye, angle of mandible and outer corner of the mouth, preoperatively, and on the second and seventh postoperative days (Fig. 9). The difference between baseline and each postoperative day indicates the level of facial swelling for that day.

## Results

A total of 20 implants were followed for 3 months postsurgically. Ten of these patients were placed using conventional flap technique and ten were placed using flapless technique. The mean age of the patient was 33.6 ± 8.4 years in conventional flap group and 46 ± 13.4 years in flapless group ranging from 25 to 60 years. Of the total patients, 60% were female and 40% male. All the 20 implants were placed in mandible (17 molars and three premolars).

**Fig. 10:**
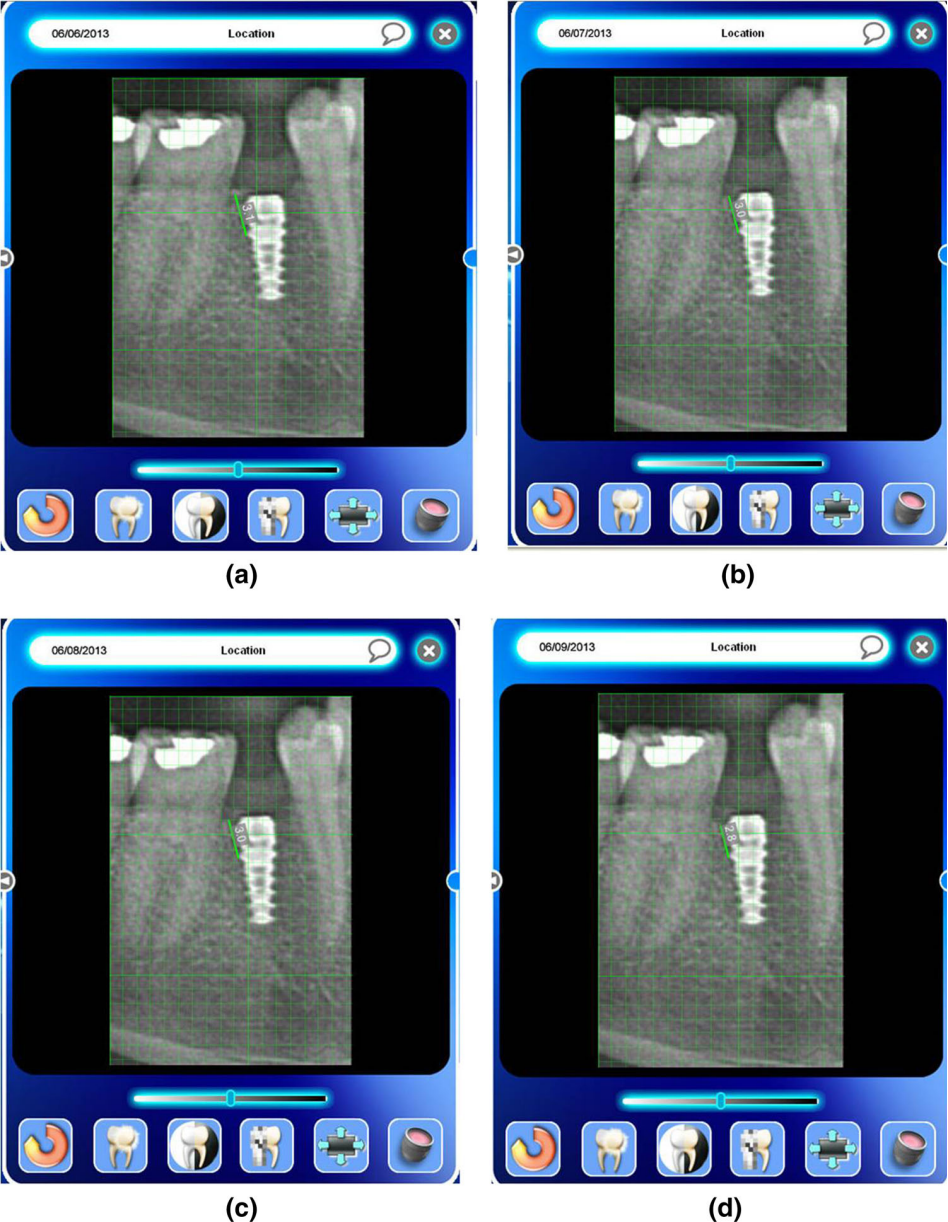
**a** Marginal bone loss assessment—conventional flap group—baseline. **b** Marginal bone loss assessment—conventional flap group—first month. **c** Marginal bone loss assessment—conventional flap group—second month. **d** Marginal bone loss assessment—conventional flap group—third month

**Fig. 11:**
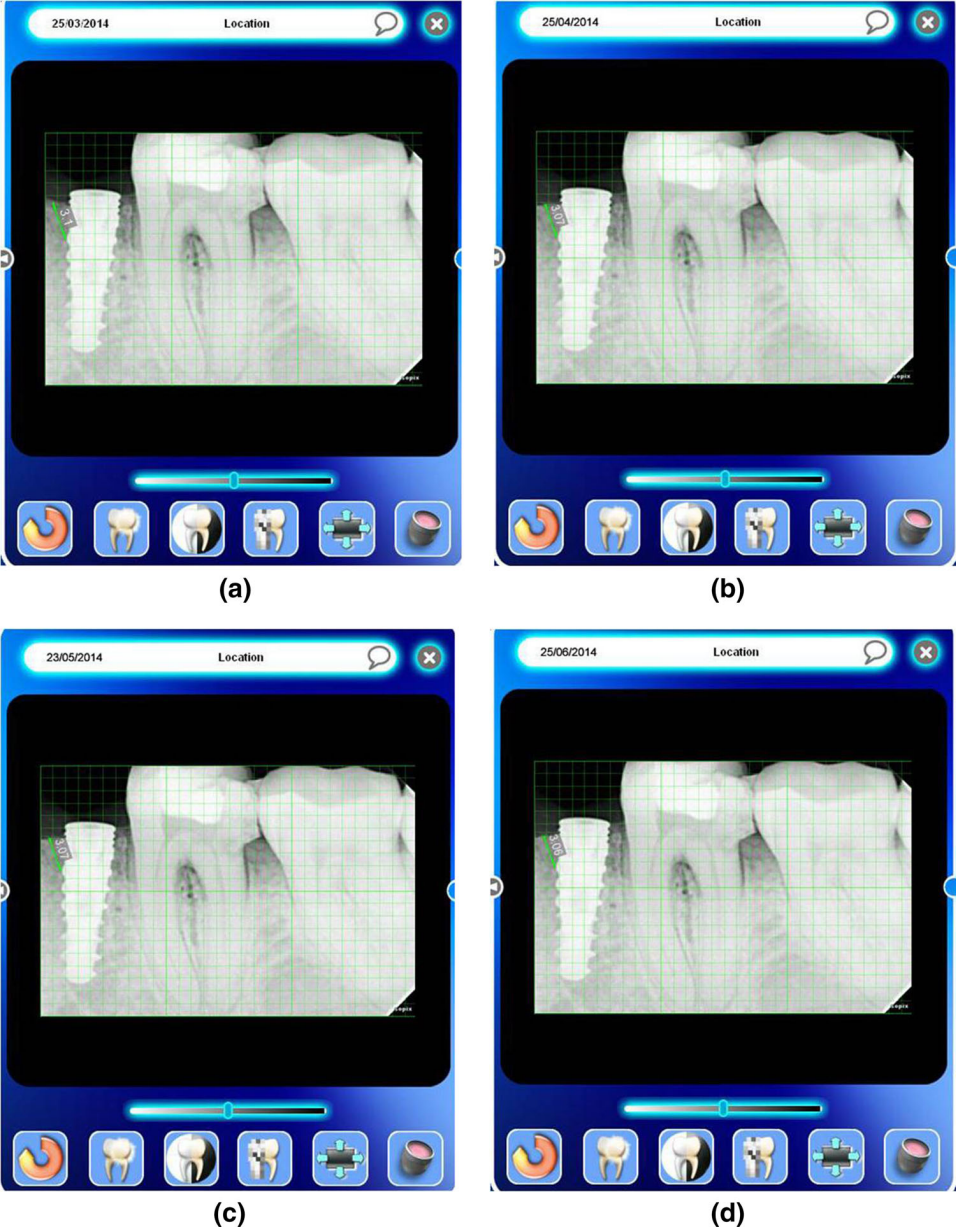
**a** Marginal bone loss assessment—flapless group—baseline. **b** Marginal bone loss assessment—flapless group—first month. **c** Marginal bone loss assessment—flapless group—second month. **d** Marginal bone loss assessment—flapless group—third month

Statistically there was significant bone loss in conventional flap group when compared with flapless group in all the time intervals. The mean difference in the bone loss for baseline to 1-month time period for flap group was 0.16 ± 0.05(Fig. 10a, b) and for the flapless group was 0.02 ± 0.005 (*p* = 0.001***) (Fig. 11a, b). The mean difference in bone loss for baseline to the second month was 0.22 ± 0.08 for flap group (Fig. 10a, c) and for the flapless group was 0.03 ± 0.005 (*p* = 0.001***) (Fig. 11a, c). The mean difference in the bone loss for baseline to the third month was 0.34 ± 0.05 for flap group (Fig. 10a, d) and for the flapless group was 0.03 ± 0.004 (*p* = 0.000***) (Fig. 11a, d). The mean difference in the bone loss for the first month to the second month was 0.06 ± 0.05 for flap group and 0.01 ± 0.01 for flapless group (*p* = 0.05*). The mean difference in bone loss for the first month to the third month was 0.18 ± 0.08 for flap group and 0.01 ± 0.005 for flapless group (*p* = 0.002**). The mean difference in bone loss for the second to the third month was 0.12 ± 0.1 for flap group and 0.004 ± 0.005 for flapless group (Table 1).

**Table 1:** Mean difference of marginal bone loss at different time intervals

Time	Group	Mean	SD	*t* value	*P* value
Baseline to 1 month	Flapless	.0240	.00548	−5.525	.001***
	Flap	.1600	.05477		
Baseline to 2 months	Flapless	.0340	.00548	−4.960	.001***
	Flap	.2200	.08367		
Baseline to 3 months	Flapless	.0380	.00447	−12.288	.000***
	Flap	.3400	.05477		
1 month to 2 months	Flapless	.0100	.01000	−2.008	.050*
	Flap	.0600	.05477		
1 month to 3 months	Flapless	.0140	.00548	−4.427	.002**
	Flap	.1800	.08367		
2 months to 3 months	Flapless	.0040	.00548	−2.365	.046*
	Flap	.1200	.10954		

Pain assessment by visual analog scale is statistically significant in all the 5 postoperative days. The mean VAS score for the first day was 5.2 ± 0.44 in flap group and 2.8 ± 0.83 in flapless group (*p* = 0.000***). The mean VAS score for the second day was 4.2 ± 0.44 in flap group and 1.6 ± 0.54 in flapless group (*p* = 0.000***). The mean VAS score for the third day was 2.8 ± 0.83 in flap group and 1.0 ± 0.00 in flapless group (*p* = 0.001***). The mean VAS score for the fourth day was 2.2 ± 0.44 in flap group and 0.0 ± 0.00 in flapless group. The mean VAS score for the fifth day was 0.8 ± 0.83 in flap group and 0.00 ± 0.00 in flapless group (Table 2).

**Table 2:** Mean VAS between flapless and conventional flap group

Postoperative days	Group	Mean	SD	*t* value	*P* value
VAS—day 1	Flap	5.2000	.44721	5.657	.000
	Flapless	2.8000	.83666		
VAS—day 2	Flap	4.2000	.44721	8.222	.000
	Flapless	1.6000	.54772		
VAS—day 3	Flap	2.8000	.83666	4.811	.001
	Flapless	1.0000	.00000		
VAS—day 4	Flap	2.2000	.44721	11.000	.000
	Flapless	.0000	.00000		
VAS—day 5	Flap	.8000	.83666	2.046	.000

There was no statistically significant value in mean differences of swelling. The mean swelling for flap group in baseline was found to be 33.26 ± 1.34 in flap group and 34.34 ± 1.7 in flapless group. The mean value for the second postoperative day was found to be 34.62 ± 1.4 in flap group and 34.78 ± 1.7 in flapless group. The mean value for the seventh postoperative day was found to be 33.46 ± 1.3 and 34.34 ± 1.7 in flapless group (Table 3).

**Table 3:** Mean swelling assessment between flapless and conventional flap group

Parameter	Group	Mean	SD	*t* value	*P* value
Baseline	Flap	33.2600	1.34462	− 1.113	.298
	Flapless	34.3400	1.70235		
Day 2	Flap	34.6200	1.42021	− .160	.877
	Flapless	34.7800	1.72250		
Day 7	Flap	33.4600	1.32023	− .913	.388
	Flapless	34.3400	1.70235		

The number of analgesics taken between the groups was highly significant in all the 5 postoperative days except on the day of surgery in which both the groups have taken equal number of tablets. The mean number of analgesics taken on first day (D1) was found to be 3.0 ± 0.00 for flap group and 1.8 ± 0.44 in flapless group (*p* = 0.000***). The mean number of analgesics for D2 was found to be 3 ± 0.0 in flap group and 1.4 ± 0.00 in flapless group (*p* = 0.000***). The mean number of analgesics for D3 was found to be 3.0 ± 0.00 in flap group and 1.0 ± 0.00 in flapless group (*p* = 0.000***). The mean number of analgesics for D4 was found to be 2.2 ± 0.44 for flap group and 0.00 ± 0.00 for flapless group (*p* = 0.000***). The mean number of analgesics for D5 was found to be 1.8 ± 0.44 for flap group and 0.00 ± 0.00 in flapless group (*p* = 0.000) (Table 4).

**Table 4:** Use of analgesic between flapless and conventional flap group

Parameter	Group	Mean	SD	*t* value	*P* value
Day 1	Flap	3.0000	.00000	6.000	.000
	Flapless	1.8000	.44721		
Day 2	Flap	3.0000	.00000	6.532	.000
	Flapless	1.4000	.54772		
Day 3	Flap	3.0000	.00000	11.000	.000
	Flapless	1.0000	.00000		
Day 4	Flap	2.2000	.44721	9.000	.000
	Flapless	.0000	.00000		
Day 5	Flap	1.8000	.44721	7.000	.000
	Flapless	.0000	.00000		

## Discussion

Management of edentulous spaces has been revolutionized by dental implants. Dental implant therapy has replaced most of the conventional methods of treating edentulous patients and has become a highly predictable treatment modality. Albrektsson et al. [4] in 1986 proposed certain criteria to assess success of implants. According to these criteria, bone loss of less than 0.2 mm annually following the implant’s first year of function is stated as being essential for long-term success [4]. Since then, the crestal bone area has been considered as a significant indicator of implant health. With the rapid advancement of dental implant therapeutics, the current trend is now geared toward enhancing esthetics and patient comfort. Establishing intact papillae and gingival contour around implants is of utmost importance, especially in patients who display soft tissue during function, such as speaking and smiling.

Branemark established the use of extensive surgical flaps to visualize the surgical field during implant surgery [5]. In the early 1970s, studies demonstrated a correlation between flap elevation and gingival recession, as well as bone resorption around natural teeth [6]. Furthermore, there has been a report of postsurgical tissue loss from flap elevation, implying that the use of flap surgery for implant placement may negatively influence implant esthetic outcomes, especially in the anterior maxilla [7]. Over the past 30 years, flap designs for implant surgery have been modified, and more recently, the concept of implant placement without flap elevation and exposure of the bony tissues was introduced. Flapless procedures have already been used for some time with tooth extractions and site preservation and have shown less morbidity. In addition, surgeons have also considered a flapless approach for immediate implants in order to preserve the vascular supply and existing soft tissue contours. Surgeons use either rotary instruments or a tissue punch to perforate the gingival tissues to gain access to bone.

When teeth are present, blood supply to the bone comes from three different paths: from the periodontal ligament, from the connective tissue above the periosteum and from inside the bone [6]. When a tooth is lost, blood supply from the periodontal ligament disappears, so that blood now comes only from soft tissue and bone. Cortical bone is poorly vascularized and has very few blood vessels running through it, in contrast to marrow bone. When soft tissue flaps are reflected for implant placement, blood supply from the soft tissue to the bone (supraperiosteal blood supply) is removed, thus leaving poorly vascularized cortical bone without a part of its vascular supply, prompting bone resorption during the initial healing phase.

The crestal bone area is considered a significant indicator of implant health [8]. Crestal bone is the area that bears the maximum stress around an implant. Blood supply to the crestal bone area is reduced around an implant compared with that of a natural tooth, because the blood vessels from the periodontal ligament are absent. Its major source of blood supply is from the periosteum covering the bone. Several studies have shown that mucoperiosteal flap elevation leads to bone resorption; however, there are few studies comparing crestal bone height between flapless and conventional flap technique.

The results of this study show that the mean difference in the bone loss for baseline to the third month for the flap group was 0.34 ± 0.05 and for the flapless group was 0.03 ± 0.004 (*p* = 0.000***). The results of the present study appear to concur with the findings of Tonetti and Schmid [9], as the cases treated with the flapless technique have shown significantly less bone loss compared with the cases treated with a conventional flap technique. Shamsan et al. [10] reported a mean crestal bone loss of 0.45 ± 0.22 mm in the flapless technique and 0.82 ± 0.09 mm in the conventional flap group. Gomez and Roman [11] supported the results of the present study by reporting that whenever it comes to marginal bone, higher bone loss rates usually occur with widely mobilized surgical flap sites where the interdental bone in the proximity to the implant is denuded from the periosteum thus affecting the nutrition of the bone and papillae, thus resulting in unpredictable degree of resorption of the interproximal marginal bone. Bhavita et al. [12] in their study showed that the overall average crestal bone resorption was 0.046 ± 0.008 on mesial aspect, 0.043 ± 0.012 on distal aspect with flapless technique, 1.48 ± 0.085 on mesial aspect and 1.42 ± 0.077 on distal aspect using with “open flap” technique. Sunitha and Sapthagiri [13] found that flapless surgery resulted in the nonsignificant crestal bone loss of (0.03–0.09 mm) on both proximal aspects during the healing period and after loading. Jeong et al. [14] observed mean marginal bone loss ranging from 0.0 to 1.1 mm with flapless technique over a period of 1 year. Becker et al. [15] also noted nonsignificant bone loss around implants placed with flapless technique until 2 years. Wood et al. [16] reported bone loss ranging from 0.23 to 1.60 mm in 4–6 months following flap elevation. Campelo and Camara [17] reported that bone resorption after using “with flap” technique is related to the thickness of the flap at the surgical site.

Job et al. [18] observed a crestal bone loss of 0.06 mm with “flapless” technique and 0.4 mm “with flap” technique over a period of 3 months. Nickenig et al. [19] found that radiographic evaluation of marginal bone levels adjacent to implants showed comparable results with flapless (0.7–2.4 mm) and flap surgery (2–3 mm) during the healing period. Similar findings were also reported by Al-Juboori et al. [20]. Jeong et al. [21] conducted their study in dogs, and after a healing period of 8 weeks they noted greater peri-implant bone height (10.1 mm) with flapless technique than at open flap site (9.0 mm). The cumulative success rate for implants placed using a flapless one-stage surgical technique varied from 74.1% to 100% after a 10-year period in a retrospective analysis done by Campelo and Camara [17]. In contrast, Pisoni Luca et al. [22] observed that there were no statistical differences in peri-implant bone resorption between the two groups, both at the basal record, implant loading and 3-year control. In our experience, there is definite advantage of flapless implant placement over the conventional flap technique in preserving the crestal bone loss. This is mainly because the crestal bone area which determines the implant health mainly depends on the periosteal blood supply [8]. Once the periosteum is stripped, there is definite loss of blood supply to the crestal bone area resulting in accelerated bone loss.

Studies quote several other advantages of flapless implant surgery, including preservation of circulation, soft tissue architecture and hard tissue volume, decreased surgical time and accelerated recuperation, allowing the patient to resume normal oral hygiene procedures immediately after the procedure [8]. In our present study, clinically patients in the flapless group have better comfort, painless postoperative days and early return to their routine day-to-day life as stated above.

Fear of pain is one of the most commonly cited anxieties associated with dental treatment. In particular, oral surgical procedures, including implant insertion, have been reported by patients to be among the most stressful and anxiety-provoking procedures in dentistry. Indeed, pain is a common complaint following dental implant surgery.

Despite the importance of pain during oral surgery for the patient and the clinician, there are few studies on the pain experienced following the placement of dental implants. Most studies fail to evaluate the intensity of pain and inflammation after surgery, and none have yet compared the patient’s perceived pain between different surgical alternatives. To evaluate the pain felt by patients, the current study used a VAS [9] which is the most widely used pain measurement instrument in many centers. The VAS is a simple, solid, sensitive and reproducible tool for assessing pain in a given patient at different points in time.

Flapless implant surgery is considered to offer advantages over the traditional flap approach, since bleeding is minimized, surgical time is shorter, and patient pain is reduced. However, studies contrasting patient outcome variables in support of these assumptions are lacking. Only one comparison has been made of flapless versus conventional flapped implant placement [23]. Therefore, the present study sought to explore patient pain/discomfort, using a subjective visual analog scale (VAS) to compare dental implant placement achieved by means of an atraumatic flapless technique with placement done with a conventional full-thickness flap technique.

The results of this present study show that there is a significant decrease in the VAS score of flapless group when compared with conventional flap with the difference being highest in the second postoperative day. Also the results of this present study show that the number of patients who felt no pain was also higher in the flapless group. These results are concurrent with studies by Shamsan et al. [10] who reported statistically significant higher mean pain severity and duration in conventional technique of implant placement compared to the flapless procedure.

The pain was also assessed by comparing the total number of analgesics taken between flapless and conventional flap group. The results of this study show that there is no painkiller taken by patients in the flapless group on the fourth day and fifth day. Except for the day of surgery, all the other postoperative days in flapless group had taken less number of analgesics when compared with conventional flap group. In accordance with the current study, Fortin et al. [24] also found that pain decreased faster and the number of patients who felt no pain was more in the flapless technique. They suggested that the objective of the flapless procedure is to reduce the invasiveness of surgery thereby reducing the surgical outcomes such as pain, edema and hematoma. This generally agrees with results reported by Chang et al. [25].

In the present study, the swelling assessment was done by the level of facial swelling which was determined by a modification of tape measuring method described by Gabaka and Matsumara [3]. The results of this present study show that there is no statistical difference in the level of swelling between these two groups. To our knowledge, there is no literature on swelling assessment in comparison between flapless and conventional flap techniques. Even though there is no statistical significance, the second postoperative day assessment value clearly shows that there is more swelling in the conventional flap group from their baseline value when compared with the flapless group.

Preoperative preparation is a critical component of the successful placement of implants using the flapless method [26]. Careful examination and diagnosis of the implant site, with radiographic assessment, is mandatory. Preoperative preparation may also include the use of computer tomography and sophisticated diagnostic software and the fabrication of a surgical template with a drilling guide for each implant.

There is a learning curve associated with every surgical procedure, after which it becomes routine. Appropriate case selection, meticulous planning, systematic surgical protocols and operator experience are required for success in flapless surgical techniques.

## Conclusion

Flapless implant surgery has many advantages and also certain disadvantages. While contemplating the use of flapless implant surgery based on this study, we should keep in mind that the cases selected for this study were ideal in terms of quantity of bone and soft tissue biotype. There is significant mean difference in the bone loss for baseline to the third month, suggesting the clinical advantage of flapless implant surgery over the conventional technique. The assessment of pain by VAS and number of analgesics suggest that there is significantly less pain in flapless implant surgery when compared with conventional flap technique.

Within the limitations of this study, it can be concluded that flapless implant surgery results in lesser loss of marginal bone and also results in better patient comfort when compared with the flap technique, provided that proper patient selection is essential for carrying out flapless implant surgery.
